# 2015, big data in healthcare: for whom the bell tolls?

**DOI:** 10.1186/s13054-015-0895-8

**Published:** 2015-04-02

**Authors:** Sven Van Poucke, Michiel Thomeer, Admir Hadzic

**Affiliations:** Department of Anesthesiology, Intensive Care, Emergency Medicine, Pain Therapy, Ziekenhuis Oost-Limburg, Schiepse Bos 6, 3600 Genk, Belgium; Department of Respiratory Medicine, Ziekenhuis Oost-Limburg, Schiepse Bos 6, 3600 Genk, Belgium; Department of Anesthesiology, Ziekenhuis Oost-Limburg, Schiepse Bos 6, 3600 Genk, Belgium

The health care sector generates bountiful data around the clock, which can paradoxically complicate our quest for information, knowledge, and ‘wisdom’ [1]. It may be prudent that medical end-users consider seriously a fundamental change that would allow us to gain full value from the ‘big data’ that the health care section is generating [2]. Proponents of the big data revolution suggest that the value for physicians rests on the added information provided by big data analysis. Indeed, supplementary information could clarify areas for improvement, such as optimization of treatments, reduced adverse events and readmission rates, earlier identification of those patients whose health is worsening, and more efficient identification of populations in need. Recent cloud computing has even turned computing and software into commodity services, and such big data processing seems to be forging a technology revolution [3,4]. However, opponents of the big data revolution speculate that validation and impact analyses of big data in health care are still in their infancy, and approaches such as Google’s baseline study may thus not be effective in preventing disease, and possibly even lead to unnecessary, if not harmful, interventions [5].

The value of any kind of data is greatly enhanced when it exists in a form that allows for integration with other data [6]. One problem with large data sets in general is the risk for ‘GIGO’ - garbage in, garbage out - that requires very careful and thoughtful investigation to rule out the many errors of large-scale data capture before any of it can be used. Thus, an essential step for data integration is the annotation of multiple bodies of data using common controlled vocabularies or ‘ontologies’ that incorporate accurate representations of biological reality [7]. Data mining in health care is not new, and initiatives for data acquisition and analysis, storage and retrieval have all been presented before [8,9]. Yet, to our knowledge, subcommittees addressing ontology have not been established by any medical specialty.

As clinicians, we apply general principles of risk stratification and risk modification to individual patients based on our education and experience. The proliferation of biomedical research makes it difficult to keep abreast of current knowledge, so clinical decision support technologies that are based on data mining techniques are knocking at our doors. Although their implementation seems inevitable, the lack of standardization continues [9]. A dramatic paradigm shift toward controlled ontologies is needed in order to optimize the technologies that integrate big data into medical decision making and practice.
